# The unusual abundance of a rare finding!

**DOI:** 10.1002/ccr3.2291

**Published:** 2019-07-30

**Authors:** Rahaf Altahan, Areej Al Mugairi

**Affiliations:** ^1^ Division of Hematopathology, Department of Pathology and Laboratory Medicine King Abdul Aziz Medical City Riyadh Saudi Arabia

**Keywords:** erythrophagocytosis, HDFN, hemolytic anemia, hemolytic disease of the fetus and newborn

## Abstract

We present images (Figure 1A‐D) of a preterm girl who had severe anemia due to HDFN caused by maternal anti‐C and anti‐D alloantibodies. These images show erythrophagocytosis, which is a very interesting and rarely encountered feature. We believe that physicians taking care of such patients should be more aware of this characteristic yet under‐detected finding.

The presence of erythrophagocytosis in the peripheral blood is rare and can easily be overlooked, and until now, only few sporadic reports describing it are present with the largest study done in 1950.^1^, ^2^ We present some elaborative blood smear images of a newborn with HDFN caused by maternal Rh‐related alloantibodies. (Figure 1A‐D).

**Figure 1:**
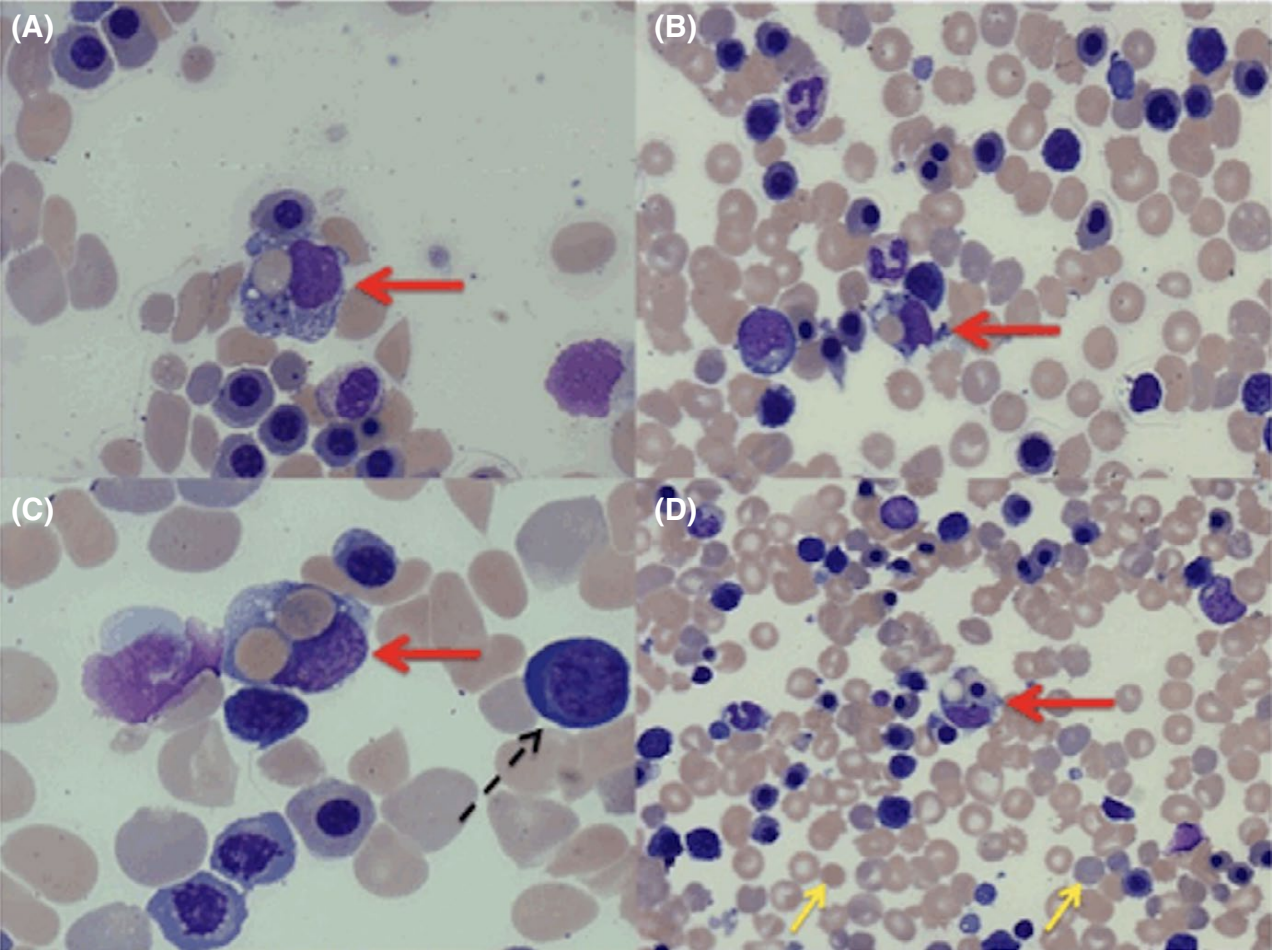
Peripheral blood smear showing significant number of nucleated red blood cells, with some erythroblasts seen (dotted black arrow). Additionally, red blood cells show moderate polychromasia and mild spherocytosis (small yellow arrows, D). Moreover, many histiocytes (bold red arrows) showing engulfed red cells (singleton: A, B; duplets: C), with fewer showing nucleated red cells (D) in their cytoplasm. Occasional left‐shifted myeloid precursors are also noted

## CONFLICT OF INTEREST

None declared.

## AUTHOR CONTRIBUTIONS

RA: involved in literature review and paper writing. AAM: involved in literature review, image acquisition, and supervision of the paper writing.
